# Commencement

**Published:** 1881-08

**Authors:** 


### COMMENCEMENT.

At the general commencement at the University of Michigan, June 30th, the degree of D. D. S. was conferred upon G. W. Avery, of Minnesota; Harry Edgerton, of Ohio; W. F. Hunt, Ohio; C. D. Peck, Ohio; R. M. Putnam, Illinois; A. E. Smith, New York; F. D. Wilson, Ohio.

There were from all the departments of the University about two hundred and fifty graduates.

The difficulties that have embarrassed the University for several years have passed away, and now there is every indication that a bright and prosperous future is before it in all departments.

